# Comparison of Knowledge about Smoking and Passive Smoking and Urinary Cotinine Levels in Pregnant Women and their Partners in Mongolia: A Cross-sectional Study

**DOI:** 10.31372/20190401.1032

**Published:** 2019

**Authors:** Naoko Hikita, Megumi Haruna, Masayo Matsuzaki, Emi Sasagawa, Minoru Murata, Ariunaa Yura, Otgontogoo Oidovsuren

**Affiliations:** a Department of Midwifery and Women’s Health, Division of Health Sciences & Nursing, Graduate School of Medicine, The University of Tokyo, Tokyo, Japan; b Department of Children & Women’s Health, Division of Health Science, Graduate School of Medicine, Osaka University, Osaka, Japan; c Mito Saiseikai General Hospital, Ibaraki, Japan; d Darkhan-Uul General Hospital, Darkhan-Uul, Mongolia; e Pharmaceutical Department, Mongolian National University of Medical Sciences, Darkhan-Uul Medical School, Darkhan-Uul, Mongolia

**Keywords:** health education, smoking prevention, smoking, passive smoking, pregnant women

## Abstract

This study aimed to compare knowledge about smoking, including passive smoking, and urinary cotinine (UC) levels in pregnant women and their partners in Mongolia. The study was conducted between November 2015 and January 2016 in Darkhan-Uul Province, Mongolia. Pregnant women with less than 20 weeks’ gestation were recruited, and their partners were also invited to participate. Self-administered questionnaires and urine samples were used to collect data. Knowledge about smoking including passive smoking was measured using 14 questions. Data were analyzed using a Student’s *t*-test, a chi-squared test, a one-way analysis of variance, and the Tukey–Kramer method for post-hoc analysis. Correlations were measured by computing Pearson’s *r* or Spearman’s *ρ*. A total of 508 pregnant women and 227 partners participated in this study; of these, 221 couples’ data were analyzed. Pregnant women’s scores on knowledge about smoking and passive smoking were significantly higher than those of their partners (9.4 ± 2.9 and 8.7 ± 3.1, respectively; *p* = 0.017). Pregnant women’s and their partners’ scores were slightly correlated (*r* = 0.163, *p* = 0.015). Pregnant women’s and their partners’ UC levels were significantly correlated (*ρ* = 0.250, *p* < 0.001). This study is the first to examine knowledge about smoking and passive smoking and UC levels among pregnant women and their partners in Mongolia. Because pregnant women’s and their partners’ scores and UC levels were positively correlated, health education on the harm caused by smoking and passive smoking should be provided to both pregnant women and their partners.

## Background

In Mongolia, it has been reported that 48.0% of men and 6.9% of women are smokers, and 42.9% of people are exposed to secondhand smoke (SHS) at home (World Health Organization, 2009). Smoking and passive smoking are harmful to one’s health: they increase the risk or exacerbate the severity of cancer, respiratory diseases, and cardiovascular diseases (U.S. Department of Health and Human Services, 2014). Smoking and passive smoking during pregnancy harm the women and fetuses; maternal smoking increases risks for ectopic pregnancy, premature rupture of membranes, abruptio placentae, miscarriage, stillbirth, preterm birth, low birth weight, and so on (World Health Organization, 2013). Passive smoking during pregnancy has been shown to increase the risks of pre-eclampsia (Luo et al., 2014), foetal congenital malformation (Leonardi-Bee, Britton, & Venn, 2011; Salmasi, Grady, Jones, McDonald, & Knowledge Synthesis Group, 2010), stillbirth (Leonardi-Bee et al., 2011), small size for gestational age (Lee, Lee, Lee, Paek, & Lee, 2015; Leonardi-Bee, Smyth, Britton, & Coleman, 2008), infants’ low birthweight (Leonardi-Bee et al., 2008; Windham, Eaton, & Hopkins, 1999), and sudden infant death syndrome (US Surgeon General, 2006).

A previous study reported that persons with more knowledge of the effects of tobacco on health are associated with greater likelihood of tobacco cessation (Chow et al., 2017; Yang, Hammond, Driezen, Fong, & Jiang, 2010). Another study reported that employers with a higher level of knowledge of smoking are more likely to promote smoking cessation in the workplace (Wang et al., 2017), while pregnant women who have less knowledge about passive smoking are significantly more likely to be exposed to SHS (Norsa’adah & Salinah, 2014; Yang, Tong, Mao, & Hu, 2010). Considering these results, knowledge about smoking and passive smoking could be an important factor in improving people’s health outcomes, especially during pregnancy. This is because pregnancy is considered to be a “teachable moment,” which is the ideal time to modify and improve lifestyle or behavior (McBride, Emmons, & Lipkus, 2003).

In Mongolia, there has been no study investigating knowledge about smoking and passive smoking that has targeted pregnant women and their partners. To reduce passive smoking in pregnant women, it is necessary to involve their family members, especially their partners, because pregnant women are often exposed to smoke involuntarily. In fact, a previous study has reported that non-smoking pregnant women’s self-awareness of SHS exposure is not accurate, compared with the levels of exposure suggested by their urinary cotinine (UC) levels (Hikita et al., 2017); cotinine is the major metabolite of nicotine, a sensitive marker of passive smoking, and widely used to assess exposure to smoke (Benowitz, 1996; Wald et al., 1984). Thus, it would certainly be possible that pregnant women are exposed to smoke unknowingly.

If we can verify that knowledge about smoking and passive smoking is related to smoking behavior, we could suggest improving health education to reduce smoking and passive smoking during pregnancy. Therefore, we believe that investigating knowledge about smoking and passive smoking among pregnant women and their partners is important to understand their present situation. Furthermore, if we can verify that pregnant women are exposed to smoke from their partners, it would be important to involve their partners in the health education classes.

The aim of this study was to compare knowledge about smoking and passive smoking and UC levels in pregnant women and their partners in Mongolia.

## Methods

### Study Design and Population

This cross-sectional study was conducted between November 2015 and January 2016 in Darkhan-Uul Province, Mongolia. Pregnant women who presented with less than 20 weeks’ gestation were recruited by trained personnel at ten public health facilities when they attended antenatal health check-ups. Their partners were also asked to participate. Pregnant women who could not understand spoken Mongolian or had difficulty in participating were excluded.

Data collection was carried out by trained medical personnel using self-administered questionnaires and analysis of urine samples. If participants had difficulty answering the questionnaires due to low literacy levels, the trained medical personnel helped them.

### Questionnaire

Socio-demographic data collected included age, educational attainment, employment status, monthly household income, number of family members, number of children living together, and information about the type of dwelling (detached house, condominium, *ger* [traditional tent-like home], or other). Participants were also asked to provide gestational age from the last menstrual period before the survey (as a re-confirmation), and their current smoking status (response options were *daily*, *less than daily*, *not at all*, and *don’t know*).

We asked a total of 14 questions about smoking and passive smoking, which were adapted from previous studies (Araki, Tanimoto, Fujii, Takato, & Yamaguchi, 1994; Nicholson, Borland, Couzos, Stevens, & Thomas, 2015), to measure participants’ knowledge. These consisted of eight True/False items and six Yes/No items, producing scores ranging from 0 to 14. The True/False items, with response options *true*, *false*, or *don’t know*, are shown in Appendix 1. The Yes/No items, with response options *yes*, *no*, or *don’t know*, are shown in Appendix 2. If participants skipped a question or selected *don’t know*, it was considered an incorrect answer, so these responses scored 0.

**Appendix 1:** True/False Questions about Smoking and Passive Smoking

Items	Correct answer, *n* (%)			
	Pregnant women		Partners	
1. Smoking doesn’t cause infertility.	109	(49.3)	101	(45.7)
2. Smoking during pregnancy increases the risk of miscarriage, premature birth, or stillbirth.	197	(89.1)	185	(83.7)
3. Smoking during pregnancy doesn’t relate to the birth of children with disability directly.	127	(57.5)	119	(53.8)
4. Toxicity from secondhand smoke is weaker than that from mainstream smoke.	132	(59.7)	123	(55.7)
5. If there is a smoker in the family, it is possible that you may have lung cancer.	165	(74.7)	152	(68.8)
6. Smoke from tobacco is not the cause of air pollution.	144	(65.2)	116	(52.5)
7. Some nutrients such as vitamins are not affected by smoking.	136	(61.5)	127	(57.5)
8. The earlier smoking initiation age is, the higher mortality rate will be.	182	(82.4)	168	(76.0)

**Appendix 2:** Yes/No Questions about Smoking and Passive Smoking

Items	Correct answer, *n* (%)			
	Pregnant women		Partners	
1. Does smoking cause lung cancer?	186	(84.2)	182	(82.4)
2. Does smoking cause heart disease?	160	(72.4)	154	(69.7)
3. Does smoking worsen diabetes?	131	(59.3)	120	(54.3)
4. Does smoking cause low birthweight?	155	(70.1)	144	(65.2)
5. Does smoking cause asthma in children from secondhand smoke?	150	(67.9)	124	(56.1)
6. Does smoking cause sudden infant death syndrome (SIDS) in children from secondhand smoke?	99	(44.8)	107	(48.4)

### Biochemical Verification of Smoking Status

In this study, we collected urine samples to verify participants’ smoking status. Participants were asked to provide urine samples after completion of the questionnaires; the samples were kept at −20 °C until analysis.

UC levels were measured using a cotinine enzyme-linked immunosorbent assay kit (Calbiotech Inc., Spring Valley, CA, USA). We requested that all UC measurement be carried out at the GYALS Medical Center, LLC in Ulaanbaatar. Absorbance was assayed using the Microplate Reader MR-96A (Shenzhen Mindray Bio-Medical Electronics Co., Ltd., Shenzhen, China) at a wavelength of 450 nm. The lower and upper limits of quantification of the kit were set at 5 ng/ml and 100 ng/ml, respectively. The intra-assay coefficient of variation was 16%, while the inter-assay coefficient was 74%.

We determined the smoking status of participants according to their UC levels regardless of their self-report. Participants with UC levels >100 ng/ml were classified as “biochemically determined smokers,” (Lee, Kim, & Lee, 2014) with 5–100 ng/ml as “passive smokers,” and with <5 ng/ml as “non-smokers.” The cut-off between passive smokers and biochemically determined smokers was set at >100 ng/ml to exclude passive smokers because previous studies reported that non-smokers’ UC levels did not exceed 100 ng/ml (Biber et al., 1987; Haufroid & Lison, 1998). The cut-off of 5 ng/ml between non-smokers and passive smokers was determined in this study because a report by the US Surgeon General concluded that even minor exposure to tobacco smoke can be harmful to health (US Surgeon General, 2006). These criteria were adopted from a previous study so that the results could be compared (Hikita et al., 2017).

### Statistical Analysis

We calculated descriptive statistics for all variables and used the chi-squared test and Student’s *t*-test to compare pregnant women’s and their partners’ characteristics. Pregnant women’s and their partners’ scores on knowledge about smoking and passive smoking were compared using the Student’s *t*-test. One-way analysis of variance, with the Tukey–Kramer method for post-hoc analysis, were performed to compare scores among three or more categories. Spearman’s rank-correlation coefficient and Pearson’s product moment correlation coefficient were used to assess the correlation. All data were analyzed using IBM SPSS Statistics 25.0 for Windows (IBM Corp., Armonk, NY, USA). Two-tailed *p*-values < 0.05 were considered statistically significant.

## Results

### Participants’ Characteristics and Smoking and Passive Smoking Knowledge Scores

A total of 508 pregnant women and 227 partners participated in this study. Of these, 15 women and three partners completed the questionnaires twice at different health facilities; therefore, we used only the responses from the questionnaires that were completed first. Furthermore, we were able to pair the responses of only 221 women with those of their partners. As such, data from 221 couples were analyzed. The questionnaire’s internal consistency, as measured with Cronbach’s alpha, was 0.721.

Table 1 shows the characteristics of 221 pairs of participants. Maternal age (mean ± SD) was 27.3 ± 5.8 years, and 120 (54.3%) pregnant women were university graduates, while partners were aged 28.6 ± 6.3 years, and 88 (39.8%) were university graduates. The number of children living at home was 1.4 ± 1.1, and the mean gestational age at recruitment was 13.1 ± 4.8 weeks.

**Table 1:** Characteristics of Participants (n = 221 Couples)

	Pregnant women		Partners		*p*
	Mean ± SD or *n* (%)		Mean ± SD or *n* (%)		
Age (years)	27.3 ± 5.8	28.6 ± 6.3	0.027
Educational attainment					0.007^a^
≤ Lower secondary school	18	(8.1)	27	(12.2)	
Upper secondary school	81	(36.7)	105	(47.5)	
≥ University	120	(54.3)	88	(39.8)	
Missing	2	(0.9)	1	(0.5)	
Employment status					<0.001^a^
Employed	89	(40.3)	117	(52.9)	
Self-employed	29	(13.1)	42	(19.0)	
Nomad	6	(2.7)	9	(4.1)	
Unemployed	94	(42.5)	49	(22.2)	
Others	0	(0.0)	1	(0.4)	
Missing	3	(1.4)	3	(1.4)	
Monthly household income^b^					
≤₮400,000	64	(29.0)	NA		
₮410,000–800,000	105	(47.5)			
≥ ₮810,000	42	(19.0)			
Missing	10	(4.5)			
Type of dwelling					
Detached house	57	(25.8)	NA		
Condominium	113	(51.1)			
*Ger*	38	(17.2)			
Others	9	(4.1)			
Missing	4	(1.8)			
Number of family members^c^	3.6 ± 1.3		NA		
Number of children living together^d^	1.4 ± 1.1		NA		
Gestational age at recruitment	13.1 ± 4.8		NA		
Self-reported smoking status					<0.001^a^
Non-smoker	201	(90.9)	84	(38.0)	
Nondaily smoker	9	(4.1)	38	(17.2)	
Daily smoker	2	(0.9)	96	(43.4)	
Missing	9	(4.1)	3	(1.4)	
UC determined smoking status					<0.001^a^
<5 ng/ml (non-smokers)	110	(49.8)	45	(20.4)	
5–100 ng/ml (passive smokers)	88	(39.8)	32	(14.5)	
>100 ng/ml (biochemically determined smokers)	23	(10.4)	142	(64.2)	
Missing	0	(0.0)	2	(0.9)	
Score of knowledge	9.4 ± 2.9		8.7 ± 3.1	0.017	

SD: standard deviation. Student’s *t*-test was used for analysis.

^a^ Chi-squared test was used for analysis. Missing data was excluded from analysis.

^b^ ₮20,000 = US$10.

^c^ Missing for 9 participants.

^d^ Missing for 24 participants.

According to participants’ self-reports, of the pregnant women, two (0.9%) were daily smokers, nine (4.1%) were nondaily smokers, 201 (90.9%) were non-smokers, and nine (4.1%) did not answer; of their partners, 96 (43.4%) were daily smokers, 38 (17.2%) were nondaily smokers, 84 (38.0%) were non-smokers, and three (1.4%) did not answer. According to participants’ UC levels, 23 (10.4%) pregnant women were biochemically determined smokers, 88 (39.8%) were passive smokers, and 110 (49.8%) were non-smokers; 142 (64.2%) partners were biochemically determined smokers, 32 (14.5%) were passive smokers, 45 (20.4%) were non-smokers, and two (0.9%) partners’ UC levels were not available.

The mean (±SD) score for knowledge about smoking and passive smoking among pregnant women was 9.4 (±2.9), while the mean score among their partners was 8.7 (±3.1), and pregnant women’s scores were significantly higher than those of partners (*p* = 0.017). Furthermore, pregnant women’s scores were significantly correlated with those of their partners (Pearson’s *r* = 0.163, *p* = 0.015; data not shown).

### Comparison of Scores on Knowledge about Smoking and Passive Smoking by Characteristics

Table 2 shows comparison of scores on knowledge about smoking and passive smoking by characteristics. The scores of pregnant women significantly differed according to the levels of educational attainment (*p* < 0.001), and those of pregnant women who had graduated from university were significantly higher than those of women who had only graduated from upper secondary school (10.2 ± 2.5 and 8.5 ± 3.1, respectively; *p* < 0.001); this pattern was not observed among men. Furthermore, the scores of pregnant women significantly differed according to the self-reported smoking status (*p* = 0.002), and those of pregnant women who identified as non-smokers were significantly higher than those of women who did not report their smoking status (9.6 ± 2.7 and 6.1 ± 4.7, respectively; *p* = 0.001). When the scores were separately analyzed in terms of active or passive smoking questions, significant differences were not seen between pregnant women who identified as non-smokers and those who self-reported that they were smokers (data not shown).

**Table 2:** Comparison of Scores on Knowledge about Smoking and Passive Smoking by Characteristics (n = 221 Couples)

	Pregnant women		*p*	Partners		*p*
	*n*	Mean ± SD		*n*	Mean ± SD	
Educational attainment^a^			<0.001			0.095
Lower secondary school	18	8.7 ± 3.4		27	8.0 ± 3.4	
Upper secondary school	81	8.5 ± 3.1^d^		105	8.5 ± 3.0	
≥University	120	10.2 ± 2.5^d^		88	9.2 ± 3.0	
Monthly household income^b^			0.132			0.120
≤₮400,000	64	8.8 ± 3.3		64	8.3 ± 3.2	
₮410,000–800,000	105	9.7 ± 2.5		105	8.5 ± 3.2	
≥₮810,000	42	9.7 ± 3.1		42	9.5 ± 2.8	
Self-reported smoking status			0.002			0.744
Non-smokers	201	9.6 ± 2.7^e^		84	8.7 ± 3.0	
Smokers	11	8.9 ± 3.5		134	8.7 ± 3.2	
Missing	9	6.1 ± 4.7^e^		3	7.3 ± 2.9	
Urinary cotinine concentration^c^			0.398			0.945
<5 ng/ml	110	9.6 ± 2.9		45	8.6 ± 3.1	
5–100 ng/ml	88	9.1 ± 3.0		32	8.8 ± 2.7	
>100 ng/ml	23	9.3 ± 2.6		142	8.8 ± 3.2	

SD: standard deviation. One-way analysis of variance were used for analysis.

^a^ Missing for 2 pregnant women and 1 partner.

^b^ Missing for 10 couples of participants.

^c^ Missing for 2 partners.

^d^ Significant difference was seen between these two groups. *p* < 0.001. Tukey–Kramer method.

^e^ Significant difference was seen between these two groups. *p* = 0.001. Tukey–Kramer method.

### Correlation between Pregnant Women’s and their Partners’ UC Levels

Table 3 shows the correlation between pregnant women’s and their partners’ UC levels. Two partners’ UC data were not available; thus, data of 219 pairs of participants were analyzed. Among pregnant women whose UC levels were 5–100 ng/ml, 71.3% of partners had UC levels >100 ng/ml, while among pregnant women whose UC levels were >100 ng/ml, 82.6% of partners had UC levels >100 ng/ml. Though the correlation coefficient was small, it is still significantly correlated (Spearman’s *r* was 0.250, *p* < 0.001).

**Table 3:** Correlation between Pregnant Women’s and their Partners' Urinary Cotinine (UC) Concentration Levels (n = 219 Couples)^a^

	Partners’ UC concentration						*p*
	<5 ng/ml		5–100 ng/ml		>100 ng/ml		
	(*n* = 45)		(*n* = 32)		(*n* = 142)		
	*n*	(%)	*n*	(%)	*n*	(%)	
Pregnant women’s UC concentration							<0.001
<5 ng/ml (*n* = 109)	37	(33.9)	11	(10.1)	61	(56.0)	
5–100 ng/ml (*n* = 87)	8	(9.2)	17	(19.5)	62	(71.3)	
>100 ng/ml (*n* = 23)	0	(0.0)	4	(17.4)	19	(82.6)	

Chi-squared test was used for analysis.

^a^ 2 partners’ UC data were not available, and thus 219 couples of participants’ data were analyzed.

Spearman’s rank-correlation coefficient = 0.250 (*p* < 0.001).

### Correlation between Pregnant Women’s UC levels and Partners’ Knowledge

Table 4 shows the correlation between pregnant women’s UC levels and partners’ scores on knowledge about smoking and passive smoking. The scores of partners whose pregnant wives’ UC levels were <5 ng/ml were significantly higher than those of partners whose pregnant wives’ UC levels were >100 ng/ml (9.1 ± 3.2 and 7.5 ± 3.3, respectively; *p* = 0.049). However, a correlation between pregnant women’s knowledge scores and their partners’ UC levels was not observed (data not shown).

**Table 4:** Correlation between Pregnant Women’s Urinary Cotinine (UC) Concentration Levels and their Partners' Scores on Knowledge about Smoking and Passive Smoking (n = 221 Couples)

		Partners’ score on knowledge	*p*
	*n*	Mean ± SD	
Pregnant women’s UC concentration			0.041
<5 ng/ml	110	9.1 ± 3.2^a^	
5–100 ng/ml	88	8.5 ± 2.8	
>100 ng/ml	23	7.5 ± 3.3^a^	

One-way analysis of variance was used for analysis.

^a^ Significant difference was seen between these two groups. *p* = 0.049. Tukey–Kramer method.

Spearman’s rank-correlation coefficient =  0.180. *p* = 0.007.

## Discussion

This study is the first to examine knowledge about smoking and passive smoking and UC levels among pregnant women and their partners in Mongolia. Pregnant women’s scores on knowledge on this topic were significantly higher than those of their partners. Furthermore, pregnant women’s scores and their partners’ scores were positively correlated. The scores of pregnant women who had graduated from university were significantly higher than those of pregnant women who had only graduated from upper secondary school. Pregnant women’s UC levels were positively correlated with their partners’ UC levels, while the knowledge scores of partners whose pregnant wives’ UC levels were <5 ng/ml were significantly higher than those of partners whose pregnant wives’ UC levels were >100 ng/ml.

### Comparison and Correlation of Knowledge about Smoking and Passive Smoking

In this study, 54.3% pregnant women and 39.8% of their partners had graduated from university. In Mongolia, women’s educational attainment is generally higher than that of men (Batsukh, Altankhuyag, & Osorgarav, 2013; Burn & Oidov, 2001), and it has been reported that 76.5% women have been enrolled in tertiary education (United Nations Educational, Scientific and Cultural Organization, 2018), and the ratio of female to male students in tertiary education is 1.4:1 (Batsukh et al., 2013). Thus, the educational level of our participants was lower than that of the general population in Mongolia, though the ratio of female to male graduates among our participants approximately reflected the national ratio.

In this study, pregnant women’s scores on knowledge about smoking were significantly higher than those of their partners. This might be due to the difference in educational levels. Furthermore, pregnant women who had graduated from university had significantly higher knowledge scores than did women who had only graduated from upper secondary school, while this pattern was not observed among men. Previous studies have reported that people with higher educational attainment obtain higher scores on knowledge on this topic (Demaio, Nehme, Otgontuya, Meyrowitsch, & Enkhtuya, 2014; Sansone et al., 2012); thus, our results support those of previous studies. Pregnant women’s consciousness of their health might be higher than that of men, because women’s perceptions of risk-reducing health behavior usually increase, and they become more careful about their health during pregnancy (McBride et al., 2003).

Self-reported non-smoking women had higher scores on smoking and passive smoking knowledge than did the women who did not answer the question about current smoking status, while no differences were found between self-reported smoking women and non-smoking women, or among male participants. We expected that people who had more knowledge of the harm caused by smoking and passive smoking would be significantly less likely to smoke, but these results were not observed in this study. A previous study has reported that some smoking pregnant women are unable to honestly report that they are smokers (Hikita et al., 2017) due to social pressure; therefore, some smokers might be included among the self-reported non-smoking women in this study. The reason women who did not answer the question regarding current smoking status had lower scores than self-reported non-smoking women might be that they were smokers and did not want to answer any questions on smoking or passive smoking because it would cause them to feel guilty.

Furthermore, there was no difference in knowledge scores between biochemically determined smokers and non-smokers (as classified with UC analysis) among either pregnant women or their partners. These results did not support the results of previous studies, which have shown that health education on the harm caused by smoking and passive smoking is effective in smoking cessation (Chow et al., 2017; J. Yang et al., 2010). One possible reason might be that the questionnaire used in this study did not provide a sufficiently sensitive measure of participants’ knowledge about smoking and passive smoking. However, the questions we used were adapted from previous studies (Araki et al., 1994; Nicholson et al., 2015), and their Cronbach’s alpha was 0.721; thus, the internal consistency of the questionnaire was acceptable. In addition, when the scores were analyzed with respect to active versus passive smoking questions, no significant differences were found between those pregnant women who self-reported that they smoked and those who self-reported that they did not smoke.

### Correlation between Pregnant Women’s and their Partners’ UC Concentration Levels

In this study, pregnant women’s UC levels were positively correlated with their partners’ UC levels. This suggests that pregnant women might be exposed to smoke from their partners. A previous study reported that pregnant women whose partners smoke were at a higher risk of SHS (Aurrekoetxea et al., 2014). However, since the correlation coefficient was small in this study, the possibility that pregnant women were exposed to smoke from people other than their partners could not be denied. Furthermore, in Mongolia, smoking in public areas such as restaurants, bars, hotels, buses, and workplaces is prohibited by law; thus, smokers tend to smoke outside and at their home. In any case, it is still essential to provide health education for pregnant women and their partners, as well as other household members, on the harm caused by smoking and passive smoking.

The knowledge scores of partners whose pregnant wives’ UC levels were >100 ng/ml were significantly lower than those of partners whose pregnant wives’ UC levels were <5 ng/ml. This result indicates that partners of pregnant wives who were biochemically determined smokers did not have enough knowledge about smoking and passive smoking to allow them to avoid its harmful effects. However, the converse result was not observed, which indicates that even if pregnant women had high levels of knowledge, it did not change their partners’ smoking behavior.

Pregnancy is a good time for women and their partners to become motivated to protect their own health and that of their fetus—in other words, it can be considered a “teachable moment” (DiClemente, Dolan-Mullen, & Windsor, 2000; McBride et al., 2003). Therefore, teaching pregnant women, their partners, and other household members about the harm of smoking and passive smoking during pregnancy is very important for increasing awareness of their own and their children’s health. Furthermore, medical personnel should promote reduction in exposure and offer smoking cessation support (World Health Organization, 2013).

### Strengths and Limitations

Above all, this is the first study to have measured pregnant women’s and their partners’ knowledge about smoking and passive smoking in Mongolia. Furthermore, this study revealed that pregnant women’s and their partners’ UC levels were positively correlated. Using biological markers such as UC in middle-income countries is somewhat difficult due to the lack of funds and measurement instruments and insufficient infrastructure. Thus, these results are extremely valuable.

Despite this strength, there are several limitations as well. First, we used only 14 items to measure participants’ knowledge about smoking and passive smoking. There were no significant differences in knowledge scores among partners according to socio-demographic differences. This might be due to the small number of questions or insufficient sensitivity of those questions. However, Cronbach’s alpha was 0.721; thus, the internal consistency of the questionnaire was acceptable. Second, because our sample was not reflective of the general population in Mongolia (educational levels of our participants were lower than those of the general population), the results cannot be generalized to the entire Mongolian population. Third, our data analysis was limited, and we could not adjust for some confounding factors. Fourth, the intra- and inter-plate assay percent coefficient of variation were fairly high, indicating that the results must be cautiously interpreted. However, as we treated UC concentration as categorical data, most inaccuracies were unlikely to affect the outcomes of the analysis.

Regarding the clinical implications of this study, our results suggest that because pregnant women’s UC levels were correlated with their partners’ UC levels, health education on the harm caused by smoking and passive smoking during pregnancy should be provided not only for pregnant women but also for their partners.

## Conclusions

This study was the first to examine knowledge about smoking and passive smoking among pregnant women and their partners in Mongolia. Scores on knowledge on this topic differed significantly between pregnant women and their partners. The scores of pregnant women who had graduated from university were significantly higher than those of women who had only graduated from upper secondary school, while such a difference was not observed among their partners. Because pregnant women’s and their partners’ UC levels were positively correlated, it is suggested that pregnant women might be exposed to smoke from their partners; thus, health education on the harm caused to their health by smoking and passive smoking should be provided for both pregnant women and their partners. In addition, individual- or group-based interventions for pregnant women with the goal of enhancing knowledge regarding the harms, susceptibility, severity of exposure to SHS, and benefits of avoiding SHS could be also effective (Chi, Sha, Yip, Chen, & Chen, 2016). Future research investigating the effects of health education on the harm caused by smoking and passive smoking for pregnant women and their partners should be conducted.

## Ethical Approval and Consent to Participate

This study was approved by the Research Ethics Committee of the Graduate School of Medicine at the University of Tokyo, Japan (No. 10934), and the Ethical Review Board of the Ministry of Health, Mongolia (No. 06, 19 November 2015). Participants were informed of the purpose and details of this study and that the results of this study would be kept confidential. Participation was emphasized as voluntary, and written informed consent was obtained from all participants. This study’s protocol complied with the principles of the Declaration of Helsinki (World Medical Association, 2013).

## Acknowledgments

We thank Battsengel Buyannemekh at the Mongolian Health Agency and Erdenetulga Buyandalai at Darkhan-Uul General Hospital for their coordination.

## Declaration of Conflicting Interests

The authors declared no potential conflicts of interest concerning the research, authorship, or publication of this article.

## Funding

This study was conducted using funds from the Japan International Cooperation Agency (JICA) Partnership Program and Research Assistant Program from the Graduate School of Medicine at the University of Tokyo.
